# Advances in mitigating methane emissions from rice cultivation: past, present, and future strategies

**DOI:** 10.1007/s11356-025-36776-8

**Published:** 2025-08-19

**Authors:** Tran Dang Xuan, Tran Thi Ngoc Minh, Ramin Rayee, Ngo Duy Dong, Nguyen Xuan Chien

**Affiliations:** 1 Transdisciplinary Science and Engineering Program, Graduate School of Advanced Science and Engineering, Hiroshima University, Hiroshima, 739-8529 Japan; 2 Center for the Planetary Health and Innovation Science (PHIS), The IDEC Institute, Hiroshima University, Hiroshima, 739-8529 Japan; 3 Faculty of Smart Agriculture, Graduate School of Innovation and Practice for Smart Society, Hiroshima University, Hiroshima, 739-8529 Japan; 4 Victory Christian Academy, 810 Buena Vista Way, Chula Vista, CA 91910 USA

**Keywords:** Methane emission, Rice cultivation, Climate change, Alternate wetting and drying (AWD), Biochar, Carbon credits

## Abstract

This paper analyzes methane emissions from rice cultivation, a major source of global methane (10–12% of emissions), driven by traditional flooding practices that create anaerobic conditions. Before 2000, continuous flooding was the dominant rice irrigation method, promoting methanogenesis and increasing methane (CH₄) emissions. Since then, practices like alternate wetting and drying (AWD), biochar application, and mid-season drainage, have significantly cut CH₄ emissions by 41.37%, 28.97%, and 23.87%, respectively. Financial mechanisms such as carbon credits, the Clean Development Mechanism (CDM), and Sustainable Rice Platform (SRP) certification now incentivize farmers to adopt low-emission techniques. These changes in water management, fertilizers, soil treatment, and policy have collectively improved methane reduction efficiency, supporting global sustainability goals. Precisions agriculture (IoT, drones, and machine learning) enabling optimized water and nutrient management. Policy mechanisms, including carbon credits and SRP certification, further incentivize sustainable practices. However, adoption faces barriers like high costs, limited training, and policy gaps. The paper also identifies future directions, including the development of stress-tolerant rice varieties, optimized microbial inoculants, and large-scale trials of AWD and IoT systems in low-income regions.

**Graphical Abstract:**

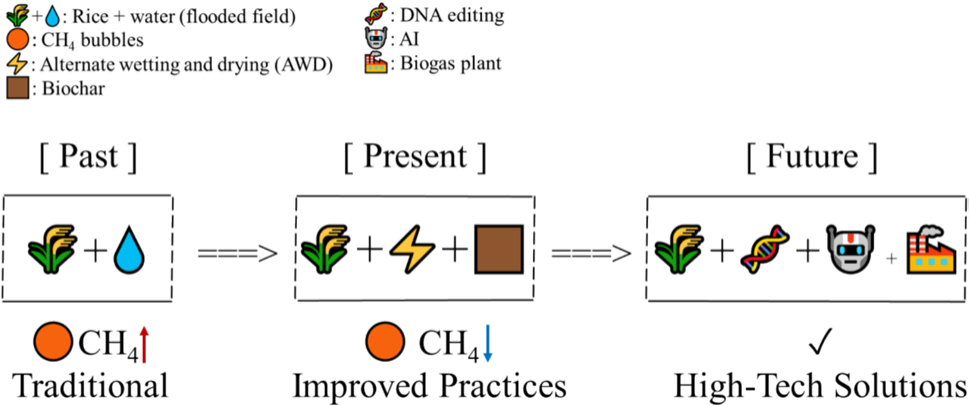

## Introduction

Rice cultivation contributes approximately 10–12% of global anthropogenic methane emissions, primarily due to anaerobic decomposition in flooded fields (Sossa et al. 2022). In Thailand, rice production accounts for nearly 60% of agricultural greenhouse gas emissions, predominantly methane (Gupta et al. 2021). In Vietnam, rice production is a major source of greenhouse gas emissions, with methane accounting for a substantial portion. A meta-analysis of 36 field sites across the country revealed that methane emission factors vary significantly across different regions and cropping seasons. The study found that average methane emission factors were higher than the default values provided by the Intergovernmental Panel on Climate Change (IPCC), indicating that emissions from Vietnamese rice production are more substantial than previously estimated (Vo et al. 2020).

Rice cultivation in the US and Japan significantly contributes to national methane emissions, prompting research into mitigation strategies. In Japan, methane emissions from rice cultivation amounted to approximately 13.07 million metric tons of CO₂ equivalent in 2022, with fluctuations observed over the decade (Statista 2022). Similarly, in the US, rice production is a major source of methane emissions. Studies have identified various factors influencing methane emissions from US rice fields, including water management practices, soil properties, and fertilizer types (Smartt 2016). Additionally, soil amendments and optimized fertilizer applications have been investigated to enhance methane oxidation and reduce emissions. For instance, incorporating organic matter during off-season periods or composting it before application can mitigate methane emissions (Yagi et al. 1997). Implementing these mitigation strategies is crucial for both Japan and the US to reduce methane emissions from rice cultivation, contributing to climate change mitigation and sustainable agricultural practices (Ahmed and Ahmed 2022).

The effective greenhouse gas (GHG) mitigation in rice paddies through early-season drainage and water-saving irrigation, reducing methane emissions by up to 90% was highlighted (Islam et al. 2018, 2020a). Strategies like reduced tillage, optimal fertilizer use, and organic methods lower GHGs and heavy metal contamination, maintaining rice yields while conserving water and resources. Reducing methane emissions from rice production is essential for meeting climate targets, as rice cultivation accounts for approximately 10% of global methane emissions (Brander et al. 2023). Implementing mitigation strategies, such as alternate wetting and drying (AWD) irrigation, can significantly decrease methane emissions by up to 48% compared to continuous flooding (Richards et al. 2022). Adopting these practices not only aids in climate change mitigation but also enhances water use efficiency and maintains or improves rice yields, contributing to sustainable agricultural development (Sander et al. 2022).

Despite advancements in mitigating methane emissions from rice cultivation, a comprehensive analysis comparing pre- and post-2000 methods is limited. Early strategies emphasized water management and fertilizer application to reduce emissions. Recent approaches incorporate innovative techniques like the application of humic acid–iron complexes. However, a detailed comparative study evaluating the evolution and effectiveness of these methods over time is lacking (Bashir et al. 2023). The Food and Agriculture Organization (FAO) has addressed methane emissions in agriculture, highlighting the need for sustainable practices in rice production. In its report, “Methane Emissions in Livestock and Rice Systems – Sources, Quantification, Mitigation and Metrics,” FAO emphasizes the importance of implementing effective mitigation strategies to reduce methane emissions from rice cultivation (FAO 2023). Additionally, studies have modeled the impact of various mitigation options on methane abatement from rice fields, offering avenues to reduce atmospheric methane (Misra and Verma 2014). Although various efforts to mitigate methane emissions in rice cultivation, there is a notable lack of comprehensive analyses comparing the evolution of mitigation methods before and after 2000. Such analyses are crucial for understanding the progression and effectiveness of strategies employed over time to reduce methane emissions in rice cultivation (Misra and Verma 2014).

In addition to the study by Misra and Verma (2014), several other studies have modeled the impact of various mitigation options on methane abatement from rice fields, offering avenues to reduce atmospheric methane. For instance, the CH4MOD model was established to estimate global methane emissions from rice paddies under different management scenarios, highlighting the potential of water management and organic matter use in mitigating emissions (Hu et al. 2024). Similarly, a report by the CGIAR Research Program on Climate Change, Agriculture, and Food Security (CCAFS) presented a global methane model for rice cropping systems, emphasizing the importance of field-specific mitigation strategies (CCAFS 2024). These studies underscore the significance of modeling in identifying effective mitigation options for reducing methane emissions in rice cultivation.

However, a detailed comparative study evaluating the evolution and effectiveness of these methods over time is lacking. Such an analysis is crucial for understanding the progression and effectiveness of strategies employed over time to reduce methane emissions in rice cultivation. This review aims to analyze and compare methane mitigation strategies in rice cultivation before and after 2000. The primary objective is to assess the evolution of water management, soil amendments, and microbial interventions in reducing methane emissions. By identifying effective practices and knowledge gaps, the review highlights the significance of sustainable rice farming in achieving global climate targets and supporting sustainable agricultural development.

### Background and mechanism of methane emission in rice cultivation

#### Methane production mechanism

In flooded rice paddies, the absence of oxygen creates anaerobic conditions that facilitate the decomposition of organic matter. This process is primarily driven by methanogenic archaea, microorganisms that produce methane as a metabolic byproduct. These archaea utilize substrates such as hydrogen and carbon dioxide to generate methane (Singh et al. 2018). The activity of methanogens is influenced by factors including temperature, pH, and the availability of organic substrates. Optimal conditions for methanogenesis typically include warm temperatures, neutral to slightly acidic pH levels, and a steady supply of degradable organic matter. Understanding the dynamics of methanogenic archaea in rice paddies is essential for developing effective strategies to mitigate methane emissions from rice cultivation (Srivastva et al. 2018). Additionally, the structure and abundance of methanogenic communities can vary between soil and root environments within rice paddies, indicating niche differentiation. This variability underscores the complexity of microbial interactions influencing methane production in these ecosystems (Ke et al. 2014). Furthermore, the coexistence patterns of soil methanogens are closely tied to methane emissions, with species interactions playing a significant role in regulating methane production. Studies measuring methane emissions across Asian rice paddies have highlighted the importance of understanding these microbial interactions to effectively manage and reduce methane emissions (Li et al. 2011).

### Sources of methane

In flooded rice paddies, methane is produced through the anaerobic decomposition of organic matter by methanogenic archaea. These microorganisms thrive in oxygen-depleted environments, utilizing substrates such as carbon dioxide and hydrogen to generate methane. The primary sources contributing to methane production include plant roots, which exude organic compounds; decomposing organic matter like rice straw; soil organic carbon; and the water column that facilitates the diffusion of organic substrates. Factors such as root development and rhizosphere soil properties significantly influence methane emissions. For instance, rice varieties with extensive root systems can enhance methane oxidation, thereby reducing emissions (Guan et al. 2024). Additionally, the spatial distribution of rice root systems affects dissolved methane concentrations in different soil layers, influencing overall methane emissions (Ding et al. 2021). Understanding these sources and their interactions is crucial for developing effective mitigation strategies to reduce methane emissions from rice cultivation.

### Factors influencing methane emissions

Methane emissions from rice paddies are influenced by several factors, including water management, soil properties, organic material input, and fertilizer type.

### Water management

The flooding of rice fields creates anaerobic conditions conducive to methane production by methanogenic archaea. Implementing intermittent drainage or alternate wetting and drying (AWD) can introduce oxygen into the soil, inhibiting methanogenesis and reducing methane emissions. It was indicated that such water management practices could significantly decrease methane emissions while maintaining rice yields (Gu et al. 2022).

### Soil properties

Soil characteristics, including texture, pH, organic carbon content, and redox potential, play a crucial role in methane emissions. Soils rich in organic matter provide substrates for methanogens, potentially increasing methane production. Conversely, soils with higher iron content can suppress methane emissions by promoting alternative electron acceptors that outcompete methanogens. Additionally, soil type and management practices largely determine the structure of bacterial communities, influencing methane emissions (Malyan et al. 2021).

### Organic material input

Rice straw can significantly influence methane emissions in paddy fields by providing more substrates for methanogenic bacteria. The degradation of rice straw in flooded conditions leads to higher methane production. However, proper management practices such as incorporating straw during the off-season, or composting it before application, can minimize methane emissions. Additionally, adjusting water management practices, such as using intermittent drainage or alternate wetting and drying (AWD), further reduces methane emissions. A study in Vietnam confirmed that integrating organic matter management with water regime adjustments not only mitigated methane but also reduced nitrous oxide emissions, showcasing the synergy between different agricultural practices to enhance sustainability (Malyan et al. 2021; Vu 2014).

### Fertilizer type

The type of nitrogen fertilizer applied in rice cultivation significantly influences methane (CH₄) emissions. Ammonium-based fertilizers, such as ammonium sulfate, have been shown to stimulate methane oxidation by methanotrophic bacteria in the rhizosphere, potentially reducing net methane emissions. This stimulation occurs because ammonium serves as a nitrogen source for methanotrophs, enhancing their activity and thereby increasing methane oxidation rates (Bodelier et al. 2000). In contrast, the effects of urea and nitrate-based fertilizers on methane emissions are more variable and depend on specific soil conditions and microbial community dynamics. For instance, urea application can lead to increased methane emissions due to the hydrolysis of urea, which raises soil pH and provides substrates that favor methanogenesis. However, in some cases, urea has been observed to have minimal impact on methane emissions, indicating that its effect is context-dependent (Lindau et al. 1991). Nitrate-based fertilizers generally tend to suppress methane production because nitrate acts as a more favorable electron acceptor for soil microbes, thereby inhibiting the activity of methanogens. However, the degree of suppression can vary based on soil characteristics and existing microbial populations (Mohanty et al. 2006).

A comprehensive study involving multi-site field observations in China found that nitrogen application rates influenced methane emissions from rice fields. The study suggested that while moderate nitrogen application could enhance rice yields without substantially increasing methane emissions, excessive nitrogen input might lead to higher emissions (Xie et al. 2010). Therefore, the selection and management of nitrogen fertilizers in rice cultivation require careful consideration of soil properties, existing microbial communities, and environmental conditions to effectively mitigate methane emissions while maintaining optimal crop yields.

In summary, methane production in flooded rice paddies occurs due to the anaerobic decomposition of organic matter, primarily facilitated by methanogenic archaea. These microorganisms utilize hydrogen and carbon dioxide to produce methane. Various factors influence methane emissions, including water management practices like alternate wetting and drying (AWD), which can reduce emissions by introducing oxygen into the soil. Soil properties such as texture, pH, and organic carbon content also play a role; soils rich in organic matter can enhance methane production, while those with higher iron content may suppress emissions. Organic material input, such as rice straw, contributes to methane emissions, but proper management techniques like composting and adjusting water regimes can reduce this impact. Fertilizer type is another crucial factor; ammonium-based fertilizers can stimulate methane oxidation, while urea and nitrate-based fertilizers have more variable effects depending on soil and microbial conditions. Effective management of these factors is essential for reducing methane emissions in rice cultivation while maintaining crop yields.

## Mitigation strategies before 2000

Before 2000, traditional rice farming practices, including water and fertilization management and organic matter handling, were significant sources of methane emissions. Continuous flooding of paddy fields created anaerobic conditions conducive to methanogenesis, with substantial methane emissions as a result (Kritee 2018). The use of urea as fertilizer exacerbated these emissions, as the ammonium ions it released stimulated methanogenic archaea (Cai et al. 2017). Furthermore, the incorporation of rice straw into flooded soils post-harvest contributed significantly to methane production due to rapid anaerobic decomposition (Linquist et al. 2012).

These practices, though essential for maintaining crop productivity, had unintended environmental consequences. However, research from recent years highlights their long-term implications and underscores the importance of sustainable alternatives. Techniques such as alternate wetting and drying (AWD) and controlled organic matter incorporation have emerged as key strategies for reducing methane emissions while sustaining rice yields.

### Conventional water management

Traditional water management in rice cultivation often involves continuous flooding, which creates anaerobic conditions in the soil. These oxygen-deprived environments favor the activity of methanogenic archaea, microorganisms that produce methane during organic matter decomposition. Methane emissions from continuously flooded rice paddies can contribute significantly to global greenhouse gas emissions. Studies estimate that rice fields account for 10–12% of global methane emissions annually (Linquist et al. 2012). While effective for weed control and stable rice yields, this practice exacerbates climate change. Sustainable alternatives, such as alternate wetting and drying (AWD), can reduce methane emissions by up to 48% (Kritee et al. 2018).

### Conventional fertilization practices

Traditional fertilization practices in rice cultivation often rely on urea as a nitrogen source. Under anaerobic conditions in continuously flooded paddies, urea hydrolyzes to release ammonium ions, which stimulate methanogenic archaea. These microorganisms utilize the ammonium as a substrate, intensifying methane production. Research indicates that urea application significantly enhances methane emissions compared to organic fertilizers (Cai et al. 1997). Additionally, excessive urea use contributes to nitrogen losses via volatilization and leaching, reducing nitrogen-use efficiency. Sustainable alternatives, such as integrated nutrient management and site-specific fertilizer application, can mitigate methane emissions and improve nitrogen utilization (Linquist et al. 2012).

### Organic matter management

Incorporating rice straw into paddy fields after harvest can enhance soil organic carbon (SOC) and nutrient cycling, as rice straw contains significant amounts of potassium, nitrogen, and phosphorus (Chivenge et al. 2019). However, this practice can also increase methane (CH₄) emissions, a potent greenhouse gas (Song et al. 2019). The timing and method of straw incorporation are crucial in mitigating these emissions. Incorporating straw in autumn, immediately after harvest, and mixing it into the soil has been shown to reduce CH₄ emissions compared to spring applications or surface spreading (Song et al. 2019). Additionally, combining straw incorporation with water management practices, such as alternate wetting and drying, can further mitigate CH₄ emissions (Ly et al. 2015). Therefore, adopting appropriate straw management strategies is essential to balance the benefits of increased SOC and nutrient recycling with the goal of reducing greenhouse gas emissions in rice cultivation.

### Crop residue management

Incorporating crop residues, such as rice straw, into paddy soils can significantly influence methane (CH₄) emissions. The decomposition of these residues under anaerobic conditions provides substrates for methanogenic archaea, leading to increased CH₄ production. However, the extent of these emissions is influenced by factors such as the amount of residue incorporated and the timing of incorporation. For instance, a study in Southwest China found that incorporating wheat residue at varying rates increased CH₄ emissions by at least 60%, with higher residue amounts leading to greater emissions (Wang et al. 2018). Additionally, the combination of residue incorporation with nitrogen fertilization can further affect CH₄ emissions. Therefore, adopting appropriate residue management strategies, such as optimizing the amount and timing of incorporation, is essential to balance the benefits of enhanced soil fertility with the goal of reducing greenhouse gas emissions in rice cultivation (Takakai et al. 2021).

### Challenges and limitations

Before 2000, efforts to mitigate carbon emissions faced significant challenges due to low public awareness, insufficient economic incentives, and weak policy frameworks. Limited understanding of climate change and its anthropogenic drivers reduced societal pressure for environmental reforms, weakening political commitment to climate action (Munasinghe et al. 2009; IPCC 2018). The absence of economic incentives, such as carbon taxes or renewable energy subsidies, further constrained the adoption of sustainable technologies (Barbier 2016). Without financial benefits or regulatory mandates, industries lacked motivation to reduce emissions (Goulder and Schein 2013). International policy mechanisms like the Kyoto Protocol were nascent, and many countries had yet to implement comprehensive climate legislation (Victor 2001). This policy vacuum enabled continued dependence on fossil fuels without accountability for environmental consequences. These factors collectively impeded the development of effective carbon mitigation strategies before the twenty-first century.

## Modern mitigation strategies (2000 to present)

### Water management innovations

Modern water management strategies have been instrumental in reducing methane (CH₄) emissions from rice paddies. Alternate wetting and drying (AWD) involves periodic drying and re-flooding of rice fields, disrupting anaerobic conditions that facilitate methanogenesis. This technique can reduce CH₄ emissions by up to 48% compared to continuous flooding (Table 1) (Malumpong et al. 2021; Dahlgreen and Parr 2024). Mid-season drainage (MSD) entails draining the field during the mid-growth stage, temporarily introducing aerobic conditions that suppress methane production. Studies indicate that MSD can significantly lower CH₄ emissions without adversely affecting rice yields (Liu et al. 2019; Martínez-Eixarch et al. 2022). Intermittent irrigation, which alternates between wet and dry periods, also reduces methane emissions by preventing prolonged anaerobic soil conditions. Compared to traditional flood irrigation, water-saving methods offer two key advantages: conserving water and increasing crop yields. In these systems, paddy fields are flooded with shallower water levels and experience longer non-flooded periods. This significantly reduces water outflow and overall water use. Field studies from 15 provinces in China show that intermittent irrigation, AWD, and winter drainage reduce CH_4_ emissions by 13–25%, 8–19%, and 30%, respectively (Ma et al. 2024). Water-saving irrigation also creates drier soil conditions, improves soil permeability, reduces nutrient loss, enhances nutrient uptake, and supports aerobic bacterial growth. However, the effectiveness of AWD relies on careful water management and may lead to increased nitrous oxide (N₂O) emissions and reduced soil carbon sequestration (Sun et al. 2022; Haque et al. 2021; Li et al. 2014). These trade-offs can partially offset the overall greenhouse gas reduction benefits in rice paddies. Therefore, implementing these practices requires careful consideration of local conditions, farmer training, and infrastructure to ensure optimal results in both yield and greenhouse gas mitigation.

**Table 1:** Effectiveness and yield impacts of various methane mitigation strategies in paddy fields

Strategy	Methane emission	Yield
AWD	− 40 to − 48% (Dahlgreen and Parr 2024; Yan et al. 2009; Zhao et al. 2024)	− 1.8–8.7% (Gao et al. 2024; Goncalves et al. 2022)
Biochar application (12 t/ha)	− 48.4% (Zhao et al. 2024; Liu et al. 2023; Sriphirom et al. 2020)	+ 7.85 to + 14.4% (Sriphirom et al. 2021; Zhang et al. 2020a, b; Qin et al. 2016)
Variety (hybrid and inbreed)	− 49.7 to − 52.3% (Zhao et al. 2024)	Depends on growth performance, biomass production, and fertilizer use efficiency
Fertilizer (60–120 kg/ha)	− 12.7 to − 52.7% (Zhao et al. 2024; Tian et al. 2015; Li et al. 2024)	+ 24.5% (Zhu et al. 2022)
Microbial inoculation	− 23.5% (Sakoda et al. 2022)	+ 52.94 (Sakoda et la., 2022)

### Soil and organic matter management

Biochar application in rice paddies enhances soil aeration and promotes methane (CH₄) oxidation, thereby mitigating greenhouse gas emissions. The porous structure of biochar improves soil porosity, facilitating oxygen diffusion and creating aerobic microsites that support methanotrophic bacteria responsible for CH₄ oxidation (Yang et al. 2024). A 2023 study demonstrated that biochar addition balanced methane emissions and rice growth by enhancing soil quality (Binh and Nghia 2023). The application of biochar in rice paddies has been shown to significantly reduce CH₄ emissions by 31.51% and increase rice yield by 7.07% (Table 1) (Wang et al. 2014). While biochar enhances soil oxygen availability, its net effect on nitrous oxide (N₂O) emissions remains uncertain. This is because improved soil moisture retention from biochar may stimulate microbial activity, potentially increasing N₂O emissions (Saarnio et al. 2013). Case et al. (2012) observed that the improved soil aeration from biochar contributed minimally to reducing N₂O emissions, suggesting that biochar influences other factors involved in N₂O production and consumption. Additionally, biochar may indirectly affect soil porosity and hydraulic properties by altering the formation and stability of soil aggregates, a process that influences greenhouse gas (GHG) emissions but remains poorly understood (Liu et al. 2012; Mukherjee and Lal 2013). Importantly, biochar’s effects on soil organic matter and CH₄ emissions are often observed over the medium to long term. Several studies have reported that biochar application improves rice yield, enhances fertilizer use efficiency, and reduces GHG emissions for up to three years (Lashari et al. 2015; Liu et al. 2022; Santos et al. 2021; Wang et al. 2023; Zhang et al. 2020a, b). Moreover, a study by Jin et al. (2024) demonstrated that a single biochar application enhanced rice yield and soil nutrient availability for six consecutive years. Conversely, incorporating fresh rice straw into flooded fields provides readily decomposable organic matter, intensifying anaerobic decomposition and increasing CH₄ emissions. Delaying straw incorporation until after the growing season or adopting off-field management practices can reduce these emissions (Belenguer-Manzanedo et al. 2022). This research also indicated that the combination of non-winter flooding and late straw incorporation strategies was effective in reducing both CH₄ and CO₂ emissions (Belenguer-Manzanedo et al. 2022). Implementing these soil and organic matter management practices is crucial for sustainable rice cultivation, balancing productivity with environmental responsibility.

### Crop and variety selection

Breeding rice varieties with shorter growing cycles and lower methane (CH₄) emissions is a promising strategy for sustainable agriculture. Recent studies have demonstrated that selecting rice genotypes with enhanced root development can significantly reduce CH₄ emissions from paddy fields, and rice varieties with robust root systems exhibited lower CH₄ emission fluxes during critical growth stages (Ding et al. 2021; Qi et al. 2024). Advancements in rice variety selection and genetic modification have further contributed to mitigating greenhouse gas emissions (Table 1). Research indicates that rice cultivar renewal, which involves introducing new varieties with improved traits, can enhance root morphology and physiology, leading to optimized photosynthate allocation and reduced CH₄ emissions. A 2022 study highlighted that such cultivar renewal strategies effectively decreased CH₄ emissions in paddy fields (Jiang et al. 2017; Hu et al. 2024; Li et al. 2011). These developments underscore the importance of integrating plant breeding and genetic approaches to develop rice varieties that not only meet yield demands but also contribute to environmental sustainability by lowering methane emissions.

### Fertilizer and nutrient management

Fertilizer and nutrient management strategies are essential for mitigating greenhouse gas emissions in agriculture. The use of ammonium-based fertilizers inhibits methanogenesis by suppressing the activity of methanogenic archaea in anaerobic soils, significantly reducing methane (CH₄) emissions from rice paddies (Table 1) (Yan et al. 2009). Additionally, slow-release fertilizers offer an effective solution to limit emissions by gradually releasing nutrients, enhancing nitrogen use efficiency, and reducing nitrogen losses to the atmosphere. These fertilizers have been shown to lower emissions of both CH₄ and nitrous oxide (N₂O) while maintaining crop productivity (Akiyama et al. 2010). Adopting these methods as part of integrated nutrient management plans can minimize the environmental impact of agricultural practices while ensuring food security. Combining these strategies with other sustainable agricultural practices provides a pathway to achieving both productivity and climate mitigation goals.

### Microbial interventions

Methane (CH₄) emissions from paddy fields are primarily driven by soil microbial activity, particularly the anaerobic production of CH₄ by methanogens and its aerobic oxidation by methanotrophs (Hussain et al. 2015). Microbial interventions are pivotal in mitigating greenhouse gas emissions in rice cultivation (Table 1). The application of methane-oxidizing bacteria, known as methanotrophs, can significantly reduce CH₄ emissions by converting CH₄ into carbon dioxide (CO₂) before it escapes into the atmosphere. Davamani et al. (2020) found that applying carrier-based methanotrophs together with nitrogen-fixing and phosphate-solubilizing bacteria to paddy fields before rice transplantation in India effectively reduced methane emissions. In rice paddies, type I methanotrophs have been identified as dominant players in methane oxidation, effectively lowering CH₄ emissions (Thao et al. 2024; Zheng et al. 2024). A 60% reduction in methane emissions and a 35% increase in rice yield have been observed following inoculation with methane-utilizing bacteria (MUB) (Davamani et al. 2020). Additionally, the composition and activity of microbial communities in anaerobic rice soils are crucial in regulating methane emissions. Methanogens, which produce methane under anaerobic conditions, coexist with methanotrophs that consume methane, creating a dynamic balance influencing overall CH₄ emissions. Understanding the interactions between these microbial communities is essential for developing effective strategies to mitigate greenhouse gas emissions from rice paddies (Li et al. 2021; Malyan et al. 2021). Implementing microbial interventions that enhance methanotrophic activity while managing methanogen populations offers a promising approach to reducing methane emissions in rice cultivation.

### Innovative technologies

Innovative technologies are revolutionizing agricultural practices, providing sustainable solutions for mitigating greenhouse gas emissions and improving water management. The integration of sensors, the internet of things (IoT), and artificial intelligence (AI) has enabled precise irrigation scheduling. These technologies monitor soil moisture, weather conditions, and crop water requirements in real-time, optimizing water use and reducing methane emissions by preventing prolonged flooding in rice paddies. AI algorithms further enhance decision-making by analyzing data patterns to determine optimal irrigation timings, ensuring that anaerobic conditions conducive to methanogenesis are minimized (Tolentino et al. 2021; Kumar et al. 2024).

Remote sensing and drone technologies are equally transformative, particularly for implementing alternate wetting and drying (AWD) practices in rice cultivation. Satellite imagery and drones equipped with multispectral cameras allow real-time monitoring of field conditions, such as soil moisture levels and vegetation indices, facilitating the effective adoption of AWD. These technologies help farmers maintain the required drying periods to disrupt anaerobic conditions, thereby significantly reducing methane emissions. Additionally, drones provide a cost-effective means of collecting detailed field data, enabling precision management of large-scale agricultural landscapes (Ramos-Fernández et al. 2024). A study by Velez et al. (2024) integrates unmanned aerial vehicle (UAV) technology equipped with multispectral sensors to monitor field conditions in rice paddies. By analyzing environmental variables and vegetation indices, the study facilitates the effective adoption of water management practices aimed at reducing methane emissions. The use of drones provides a cost-effective means of collecting detailed field data, enabling precision management across large agricultural landscapes (Velez et al. 2024). By leveraging these innovative tools, farmers can enhance resource efficiency, mitigate environmental impacts, and contribute to achieving climate resilience in agriculture.

### Policy instruments and carbon market mechanisms

Policy instruments and carbon market mechanisms play a pivotal role in promoting low-emission practices in rice farming. The Clean Development Mechanism (CDM), established under the Kyoto Protocol, enables developed countries to invest in emission reduction projects in developing nations, such as methane mitigation in rice cultivation, earning certified emission reductions (CERs) in return (Pahuja 2023). Additionally, the Sustainable Rice Platform (SRP) promotes sustainable rice cultivation through the development of standards and practices that reduce greenhouse gas emissions. Farmers adhering to SRP guidelines can enhance their marketability and potentially access carbon credit schemes, aligning economic incentives with environmental sustainability (Connor et al. 2023).

Carbon credits specifically for low-emission rice farming provide financial incentives for adopting climate-smart agricultural practices. By implementing techniques such as alternate wetting and drying (AWD) and utilizing innovative technologies, farmers can reduce methane emissions and earn carbon credits, contributing to global climate mitigation efforts (Jang et al. 2023; Xuan et al. 2025). These mechanisms not only support the reduction of greenhouse gas emissions but also promote sustainable development in the agricultural sector. Carbon credit programs reward these efforts by providing farmers with a marketable asset that generates additional income. The credits can be sold in voluntary or compliance-based carbon markets, allowing farmers to financially benefit from their sustainability initiatives. This dual benefit of economic gain and environmental impact positions low-emission rice farming as a key component in global climate mitigation strategies.

## Comparative Analysis of Before 2000 and After 2000

Over the past few decades, rice cultivation practices have evolved significantly, leading to enhanced methane (CH₄) emission reduction efficiencies (Table 2). Prior to 2000, continuous flooding was the standard irrigation method in rice farming, creating anaerobic conditions that favored methanogenesis. In recent years (Table 2), techniques like alternate wetting and drying (AWD) and mid-season drainage have been adopted. These methods intermittently aerate the soil, disrupting methane-producing microbial activity and substantially reducing CH₄ emissions. Studies have shown that AWD can decrease methane emissions by up to 48% compared to continuous flooding (Bashir et al. 2023; Islam et al. 2020a, b). The shift from traditional urea to ammonium-based fertilizers has further contributed to methane mitigation (Table 2). Ammonium-based fertilizers inhibit methanogenesis by altering soil pH and microbial communities, leading to lower CH₄ emissions. Additionally, the use of slow-release fertilizers has been shown to limit emissions by providing a steady nutrient supply, reducing the need for frequent applications (Akiyama et al. 2010; Senthilraja et al. 2023).

**Table 2:** Key differences between mitigation practices before 2000 and recent years

Category	Before 2000	2000 to present
Water management	Continuous flooding (Bashir et al. 2023; Islam et al. 2020a)	AWD, mid-season drainage (Bashir et al. 2023; Islam et al. 2020a)
Fertilizers	Urea (Akiyama et al. 2010; Senthilraja et al. 2023)	Ammonium-based fertilizers (Akiyama et al. 2010; Senthilraja et al. 2023)
Organic matter	Fresh straw incorporation (Song et al. 2019; Islam et al. 2020a; Senthilraja et al. 2023)	Straw pre-decomposition (Song et al. 2019; Islam et al. 2020a; Senthilraja et al. 2023)
Soil management	Limited biochar use (Dong et al. 2013; Lee et al. 2023)	Widespread biochar use (Dong et al. 2013; Lee et al. 2023)
Policy and market	No carbon credits (Bashir et al. 2023; Jang et al. 2023)	Carbon credits, CDM (Clean Development Mechanism), SRP (Sustainable Rice Platform) certification (Bashir et al. 2023; Jang et al. 2023)

Incorporating fresh straw into paddy fields was common practice before 2000, inadvertently increasing methane emissions due to rapid decomposition under anaerobic conditions (Table 2). The modern approach involves pre-decomposing straw before incorporation, reducing the availability of labile carbon for methanogens and thereby decreasing CH₄ emissions (Song et al. 2019; Islam et al. 2020b; Senthilraja et al. 2023). The application of biochar has become widespread in recent years (Table 2). Biochar improves soil structure and increases porosity, enhancing aeration and promoting the activity of methane-oxidizing bacteria (methanotrophs). This leads to a significant reduction in methane emissions from rice paddies (Dong et al. 2013; Lee et al. 2023).

Previously, there were no financial mechanisms to encourage low-emission farming practices. The introduction of carbon credits, the Clean Development Mechanism (CDM), and Sustainable Rice Platform (SRP) (Table 2) certification now provide economic incentives for farmers to adopt climate-smart practices. By implementing techniques like AWD and utilizing innovative technologies, farmers can reduce methane emissions and earn carbon credit, contributing to global climate mitigation efforts (Bashir et al. 2023; Jang et al. 2023; IRRI 2024). These advancements in technology, policy, and scientific understanding have collectively enhanced the efficiency of methane reduction in rice cultivation, aligning agricultural practices with global sustainability goals.

## Case studies in China, Vietnam, Thailand, and India

In 2020, methane (CH₄) emissions from rice paddies in Asia accounted for 86.53% of global rice paddy CH₄ emissions (Ma et al. 2024). Effective water management plays a central role in controlling CH₄ emissions in Southeast Asian countries, prompting the implementation of policies that promote alternate wetting and drying (AWD) techniques (Wang et al. 2022). In China, intermitted irrigation reported to decrease CH_4_ by 43.83% (Table 3). A large-scale modeling study that integrated the Decision Support System for Agrotechnology Transfer (DSSAT) with the DeNitrification-DeComposition (DNDC) model simulated AWD adoption in rice systems across China, revealing CH₄ emission reductions of 38.89% in southern regions and 60–71% in northeastern regions compared to continuous flooding (Nikolaisen et al. 2023; Li et al. 2006). As illustrated in Table 3, water management practices exhibit significant variation in their effectiveness across China and Southeast Asian countries. These differences may stem from variations in climate, soil characteristics, and rice production systems, which influence the outcomes of mitigation strategies.

**Table 3:** Changes in CH_4_ emission under different measurements across major rice production regions in Asia

Measurement	China	Vietnam	Thailand	India
Intermittent irrigation	− 43.83% (Zhao et la. 2019)		− 27.99% (Cha-Un et al. 2021)	
AWD	− 38.99% (Ma et al. 2024)	− 50% (Minamikawa et al. 2021; Vu 2014)	− 43.87% (Cha-Un et al. 2021)	− 32.62% (Oo et al. 2020)
Winter drainage	− 40.24% (Ma et al. 2024)			
Urea	− 32.49% (Ma et al. 2024; Li et al. 2018)			
Organic manure				− 15.66% (Bharali et al. 2018)
Biochar	− 31.51% (Ma et al. 2024)	− 30.5% (Nam et al. 2024)	− 24.9% (Sriphirom et al. 2021)	

Beyond AWD, China has implemented several complementary agricultural practices to reduce CH₄ emissions from soils. These include the application of water drainage and use of advanced nitrogen fertilizer management techniques such as polymer-coated controlled-release urea, urea with N-Serve (nitrapyrin), and urea with 3,4-dimethylpyrazole phosphate (CRU/NU/DMPP) which resulted in 40.24 and 32.49% CH4 reduction, respectively (Table 3).

China also promotes biochar application with obtained results of 31.51% CH_4_ reduction (Table 3) and direct seeding and plastic-film mulching as methane-reducing agricultural strategies in rice cultivation. Direct seeding reduces the duration of field flooding, thereby lowering CH₄ emissions to some extent (Ma et al. 2010). However, direct seeding is more susceptible to disease transmission compared to traditional transplanting methods, often requiring 1–2 times more herbicide applications and increasing total herbicide doses by 2–3 times (Xu et al. 2018). Plastic-film mulching, primarily intended for water conservation, replaces flooded irrigation with wet cultivation by covering the soil with plastic film (Zhang et al. 2013). Together, direct seeding and plastic-film mulching can reduce CH₄ emissions by 37.59%, although with only a marginal yield increase of 0.06% (Ma et al. 2024).

Vietnam has successfully implemented the alternate wetting and drying (AWD) technique to reduce methane emissions in rice cultivation. AWD involves periodic drying and re-flooding of rice fields, disrupting anaerobic conditions that produce methane. This method has led to a reduction in methane emissions by up to 50% in Vietnam and a decrease in water usage by approximately 30%, without compromising rice yields (Table 3). Additionally, biochar application in Vietnam has resulted in 31.5% CH_4_ reduction (Table 3). The International Rice Research Institute (IRRI) has been instrumental in promoting AWD in Vietnam, contributing to the country’s climate change mitigation efforts (Pham et al. 2021; CCAFS 2024).

In Thailand, the application of biochar in rice farming has shown promise in enhancing soil fertility and reducing greenhouse gas emissions. Biochar, a carbon-rich product obtained from biomass, improves soil properties and sequesters carbon. Studies have demonstrated that incorporating biochar into paddy soils can lead to a significant reduction in methane emissions. In Thailand, the application of AWD and biochar has resulted in CH₄ emission reductions of 43.87% and 24.9%, respectively (Table 3). Additionally, biochar application has been associated with increased rice yields, making it a viable strategy for sustainable agriculture in Thailand (IRRI 2024; Wijitkosum and Jiwnok 2019; Sriphirom et al. 2021).

India has applied application of AWD and organic manure which has resulted in CH₄ emission reductions of 32.62% and 15.66%, respectively (Table 3). Additionally, India has adopted the Sustainable Rice Platform (SRP) standards to promote sustainable rice cultivation. The SRP provides a framework for resource-efficient and climate-resilient rice production, offering farmers access to premium markets and financial incentives. By adhering to SRP standards, Indian farmers implement practices that reduce environmental impact, such as efficient water management and reduced chemical use. This approach not only contributes to global climate mitigation efforts but also enhances farmers’ livelihoods through better market access and potentially higher prices for sustainably produced rice (SPR 2022; ECOCERT 2024; IFC 2024).

## Case studies in Japan, the US, and EU

Reducing methane (CH₄) and carbon dioxide (CO₂) emissions in rice cultivation has been a focus in Japan, the European Union (EU), and the United States (US). Japan has implemented water management strategies such as the system of rice intensification (SRI), which involves intermittent irrigation to reduce CH₄ emissions. A study demonstrated that SRI reduced global warming potential, methane, and carbon dioxide emissions by 57.1%, 59.2%, and 25%, respectively, compared to conventional practices (Mboyerwa et al. 2022). The method of no-tillage rice cultivation using controlled-release fertilizers can improve soil environments and reduce greenhouse gas emissions (Harada et al. 2007).

The EU has promoted AWD (alternate wetting and drying) techniques to mitigate methane emissions from rice fields. The European Commission’s methane strategy highlights that emissions can be reduced by rewetting, drying, and other appropriate agricultural practices (EC 2020). For policy initiatives, the EU’s comprehensive strategy to reduce methane emissions includes promoting agricultural practices that lower emissions from rice cultivation. In case of the US, they have explored water management strategies, such as AWD, to reduce methane emissions in rice cultivation. The World Resources Institute discusses options like mid-season drawdowns and sequences of wetting and drying to prevent methane buildup (EC 2020). The emission reduction initiatives in the US focus on implementing practices that reduce greenhouse gas emissions from rice production, including improved water management and cultivation techniques (EC 2020). These studies and initiatives demonstrate the efforts in Japan, the EU, and the US to adopt practices that mitigate methane and carbon dioxide emissions in rice cultivation.

### Challenges and limitations

Implementing methane-reducing practices in agriculture presents several challenges across technological, economic, policy, and scientific domains. The adoption of advanced agricultural technologies is often hindered by high costs, limited access, and insufficient farmer training. For instance, implementing water-saving systems like alternate wetting and drying (AWD) requires precise control mechanisms, which can be expensive and complex to operate. Additionally, the integration of digital tools for monitoring and managing these systems necessitates technical expertise that many farmers may lack. This technological gap can impede the widespread adoption of such practices (Suwanmaneepong et al. 2023; Bashir et al. 2023).

The financial burden associated with sustainable farming practices is a significant deterrent for many farmers. The production and application of biochar, for example, involve substantial costs that may not be immediately offset by increased crop yields or carbon credits. Similarly, investing in water-saving technologies requires upfront capital that smallholder farmers might find prohibitive. Without adequate financial support or incentives, the economic feasibility of adopting these practices remains questionable (Bashir et al. 2023; World Bank 2024). Effective policy frameworks are crucial for promoting sustainable agricultural practices. Currently, there is a lack of comprehensive policies that provide incentives or funding for methane-reducing techniques. The absence of subsidies, tax breaks, or other financial incentives diminishes the motivation for farmers to transition to environmentally friendly methods. Moreover, inconsistent policy support can lead to uncertainty, further discouraging adoption (Ahmed et al. 2023; CGS 2024).

While there is growing interest in microbial inoculants to inhibit methanogenesis, research in this area is still in its infancy. Limited understanding of the interactions between introduced microbial species and native soil microbiota poses challenges in developing effective inoculants. Additionally, the long-term effects of such interventions on soil health and crop productivity remain uncertain, necessitating further investigation (Yagi et al. 1997; Ambrosini et al. 2016; Nie et al. 2024). Addressing these challenges requires a multifaceted approach, including investment in affordable technologies, financial support mechanisms, robust policy frameworks, and comprehensive scientific research. Collaborative efforts among governments, research institutions, and the agricultural community are essential to overcome these barriers and promote the adoption of methane-reducing practices in agriculture.

## Future Directions and Emerging Innovations

Advancements in agricultural technology, research, and policy are paving the way for more sustainable and efficient rice cultivation practices. These developments hold promises for enhancing productivity while mitigating environmental impacts. Breeding programs focus on developing rice varieties that are resilient to climate change, resistant to pests and diseases, and have higher nutritional value. For instance, the International Rice Research Institute (IRRI) (IRRI 2024) has been instrumental in developing stress-tolerant rice varieties suitable for various agro-ecological zones. These next-generation varieties aim to ensure food security and improve farmers’ livelihoods (Hasan et al. 2024; Rayee et al. 2024; IRRI 2024). Furthermore, emission factors related to crop management and rice variety have been suggested for modeling the impact of rice cultivation on CH_4_ emissions. However, the role of rice variety remains debated. While Kerdchoechuen (2005) found significant differences in CH_4_ emissions among four Thai rice varieties grown in sand under controlled pot conditions, Asch et al. (2023) observed only minor varietal effects compared to other factors like seasonality and fertilizer practices. More recently, Vo et al. (2023) demonstrated that among 20 Vietnamese rice varieties, seasonal methane emissions differed by approximately 40–45% between the lowest and highest emitters. In production systems where alternate wetting and drying (AWD) is not feasible, other strategies such as optimizing fertilizer use or managing soil organic matter have been considered. However, the potential of selecting low-emission rice varieties as a mitigation approach has received relatively little attention until now.

The integration of machine learning algorithms with drone technology is revolutionizing precision agriculture. Drones equipped with multispectral sensors collect high-resolution data on crop health, soil conditions, and pest infestations. Machine learning models analyze this data to provide actionable insights for farmers. For example, a study demonstrated the use of unmanned aerial vehicles (UAVs) and deep learning algorithms to predict growth stages of rice varieties, enhancing crop management strategies (Zhang et al. 2024). Microbial inoculants, such as plant growth-promoting bacteria and fungi, have shown potential in enhancing crop productivity and soil health. However, challenges remain in their large-scale application, including environmental interactions and field efficiency. Research is needed to optimize formulations and application methods to ensure consistent performance under diverse field conditions (Khare et al. 2015; dos Reis et al. 2024). Alternate wetting and drying (AWD) is a water-saving technique that can reduce methane emissions in rice paddies. Combining AWD with internet of things (IoT) technologies can enhance its effectiveness. Smallholder farmers in tropical regions are particularly susceptible to the impacts of climate change due to their constrained resources and reliance on marginal lands. AWD offers a low-cost and accessible technique that holds promise for improving their livelihoods. However, its effectiveness in alleviating poverty and enhancing resilience is closely tied to issues of social equity. Many poor farmers face barriers such as limited access to irrigation systems, infrastructure, and financial services. As a result, any programs promoting AWD or similar technologies must be tailored to address these specific challenges to prevent deepening existing social and economic disparities. Additionally, conducting large-scale trials in low-income regions is essential to assess the feasibility, scalability, and socio-economic impacts of these integrated approaches. Such studies can inform the best practices and policy recommendations for sustainable rice cultivation (Siddiqui et al. 2021; Pham et al. 2021).

Robust carbon credit systems can provide financial incentives for farmers adopting methane-reducing practices. To encourage farmers to adopt new rice management practices, even if it might lead to some yield loss (Johnson et al. 2023), a compensation system should be created. For instance, a 25% cut in CH_4_ emissions would yield 4.75 to 9.50 $ per hectare per season (Asch et al. 2023). Given an average profit of 1000 $ per ha and season from rice farming in the Mekong River Delta (Berg et al. 2017), this corresponds to an additional 2.5 to 5% income. Based on these simplified calculations and by considering additional transaction costs, the direct payment of carbon credits to farmers appears to be an inefficient strategy for increasing livelihoods. However, if these payments are aggregated at larger scale, either for entire cooperatives or by integrating individual farms, it could become an add-on in support of rural development such as investing in irrigation facilities. Strengthening these systems involves establishing clear guidelines, ensuring transparency, and facilitating market access for smallholder farmers. Effective carbon credit mechanisms can make sustainable practices economically viable and attractive (AAJ 2023; FAO 2024). In conclusion, the future of sustainable rice cultivation lies in the synergistic advancement of technology, research, and policy. Embracing next-generation rice varieties, precision agriculture tools, optimized microbial inoculants, and supportive policy frameworks can lead to a more resilient and environmentally friendly rice production system (APRI 2024; IFAD 2024).

## Conclusion

Over the years, rice cultivation has evolved to balance food security with environmental sustainability. Traditional practices like continuous flooding and fresh straw incorporation, which significantly contributed to methane (CH_4_) emissions, have been replaced by modern techniques such as alternate wetting and drying (AWD), biochar application, and straw pre-decomposition which may reduce CH_4_ emission by 41.37%, 28.97%, and 23.87%, respectively. These methods demonstrate that sustainable practices can align environmental goals with economic incentives.

Present strategies emphasize technological integration, such as IoT, drones, and machine learning, to optimize water and nutrient management. Policy instruments like carbon credits and Sustainable Rice Platform (SRP) certification incentivize low-emission practices. However, barriers like high costs, limited farmer training, and gaps in financial and policy support remain significant challenges. Future directions include stress-tolerant rice varieties and optimized microbial inoculants, offering scalable solutions for methane mitigation. Large-scale trials of AWD and IoT systems through supportive programs and cooperatives in low-income regions are crucial to ensuring feasibility and inclusivity. Collaboration among governments, research institutions, and private sectors is vital to support farmer education, financial incentives, and access to modern tools. Policies must focus on strengthening carbon credit systems and fostering international cooperation to accelerate sustainable farming transitions. Ultimately, combining innovative techniques, robust policies, and inclusive research will pave the way for a sustainable rice production system that benefits farmers, consumers, and the environment.

## Data Availability

The data presented in this study are available on request from the corresponding author.
